# Carbon Dot–Doped Titanium Dioxide Sheets for the Efficient Photocatalytic Performance of Refractory Pollutants

**DOI:** 10.3389/fchem.2021.706343

**Published:** 2021-09-07

**Authors:** Shen Shen, Rong Li, Hongbo Wang, Jiajia Fu

**Affiliations:** ^1^ Jiangsu Engineering Technology Research Centre for Functional Textiles, Jiangnan University, Wuxi, China; ^2^ Key Laboratory of Eco-textiles, Ministry of Education, Jiangnan University, Wuxi, China

**Keywords:** carbon dots, visible light, photocatalytic degradation, congo red, stability

## Abstract

Broad solar light harvesting and fast photoinduced electron–hole migration are two critical factors for the catalytic capacity of photocatalytic system. In this study, novel visible light–driven carbon dot–TiO_2_ nanosheet (CD-TN) photocatalysts are successfully prepared by loading CDs on the surface of TNs through the hydrothermal method. The microstructure, chemical components, and optical properties of the prepared samples are characterized *via* X-ray diffraction, Fourier transform infrared spectroscopy, transmission electron microscopy, UV-visible diffuse reflectance spectroscopy, and X-ray photoelectron spectroscopy analysis. Congo red (CR), rhodamine B (RhB), and tetracycline (TC) are selected as pollutants to assess the catalytic performance of CD-TNs. As expected, the removal efficiencies of CD-TNs for CR, RhB, and TC are 94.6% (120 min), 97.2% (150 min), and 96.1% (60 min), respectively, obviously higher than that of pure TNs. The enhanced degradation efficiency of CD-TNs is predominantly ascribed to the merits of CDs (excellent up-conversion property and electron transfer property). Moreover, according to the several degradation cycles, CD-TNs possess the excellent stability, having removed 93.3% of CR after 120 min irradiation.

## Introduction

Increasing awareness of aquatic contamination and environment crisis have spurred explosive research on solar energy conversion and utilization (Li et al., 2017a; Hu et al., 2018; Kayaalp et al., 2019). Wastewater is mostly generated from the spillage of a broad range of organic contaminants, which are extensively present in industrial, agricultural, and household applications (Zhang et al., 2017a; Wang et al., 2017; Shen et al., 2021). Organic reagents, closely linked with industry, are typical by-product of the rapid development of human society (Basith et al., 2018; Sordello et al., 2018). Not only effective contamination degradation but also appropriate applications of the purified water should benefit to the remediation of wastewater. However, due to their complex chemical structure, it is difficult to damage these pollutants, which are intentionally chosen to resist sunlight and oxidation by microorganisms (Zhang et al., 2017a; Saitow et al., 2018). For this reason, removal and lowering of toxicity of pollution effluents from wastewater is impending (Fan et al., 2015; Gao et al., 2018).

Organic pollutants removal through photocatalytic technology has spurred intense research in recent years, since it offers an environmentally friendly route to transfer solar energy into chemical energy for reducing the excessive pollution (Li et al., 2018a; Lu et al., 2018; Zewu et al., 2018; Liu et al., 2019a). The common photocatalyst is TiO_2_ for its fascinating features and relatively high photocatalytic capacity (Liu et al., 2019b). A bare TiO_2_, howbeit, is excited only under UV light, which attributes to its large bandgap (about 3.2 eV) (Zhang et al., 2016a). Thus, enormous efforts have been devoted to exploring the reaction processes and developing methods to improve the photocatalytic activity and efficiency of TiO_2_ (Liu et al., 2019b; Lee et al., 2019). As expected, huge breakthroughs have emerged in the process of preparation, functionalization, and modification of TiO_2_-based photocatalysts to enhance the utilization of solar spectrum (∼50% of solar light) for practical applications (Collado et al., 2018; She et al., 2019). In general, modifying TiO_2_ with appropriate dopant not only suppresses the photo-induced e^+^-h^-^ recombination rate but also increases more visible light capacity that is absent with pure TiO_2_. Various strategies have been investigated to advance activities and applications of TiO_2_, including elements doping (Han et al., 2019), surface modification (Fu et al., 2017), semiconductor heterojunction (Zhen-Dong et al., 2018), and so on (Park et al., 2019).

Carbon materials are impressive candidates due to their striking optical and superior electron-transfer properties (Wang et al., 2016), including carbon dots (CDs; Zhang et al., 2016b; Chen et al., 2017; Sang et al., 2017), carbon nanotube (Li et al., 2017b), graphene (Xia et al., 2019; Xin et al., 2019), and graphene quantum dots (Zhi et al., 2019). Among them, carbon dots with size below 10 nm possess unique merits and have attracted widespread interests to improve the photocatalytic performance of catalysts (Cheng et al., 2016; Zhang et al., 2017b). The introduction of CDs in photocatalysts can extend the absorbance of sunlight and prompt electron transfer and decomposition of photoinduced electron–hole pairs, hence enhance the photocatalytic property of catalysts (Li and Zhang, 2018).

In this study, CD-TN photocatalysts were synthesized *via* the hydrothermal method and were applied to remove the three different pollutants (CR, RhB, and TC) under visible light irradiation. The morphology structures, chemical states, and optical properties of the prepared photocatalysts were investigated in detail. The photocatalytic performance of the as-prepared photocatalysts was tested by the decomposition of CR, RhB, and TC, which are toxic to human health under visible light irradiation. Additionally, the stability of CD-TN photocatalysts was also evaluated by five successive cycles.

## Experimental

### Chemicals

Tetrabutyl titanate (TBOT), citric acid (CA), CR, and TC were purchased from Aladdin Reagent Co., Ltd. (China). Hydrofluoric acid (HF), sodium hydroxide (NaOH), and RhB were provided by Sinopharm Chemical Reagent Co., Ltd. (China). Ammonium hydroxide (NH_4_OH) was purchased from J&K Scientific Ltd. Deionized (DI) water used in the experiments was obtained using an ULUPURE pure water/water system.

### Synthesis of TiO_2_ Nanosheets

Initially, 3 ml of HF was mixed with 25 ml of TBOT and stirred for 30 min. Thereafter, the mixture was transferred to a Teflon-lined autoclave and maintained at 180°C for 24 h. After that, the obtained powders were filtered and washed with 0.1 M NaOH solution and DI water to remove the unreacted F^−^. The white products were dried at 60°C for 12 h under vacuum.

### Synthesis of CDs

1.051 g of CA was dissolved into 25 ml of DI water. NH_4_OH was added into the above solution dropwise under stirring to adjust the pH value to 6. Then, the mixture was placed into a Teflon-lined autoclave and maintained for 4 h under 160°C. Then, the solution was filtrated by centrifugation and purified by dialysis (Mw = 3,500). Afterward, the CD solution was collected and freeze-dried for further use.

### Synthesis of Carbon Dot/TiO_2_ Nanosheets

First, 1.051 g of citric acid was added into 25 ml of DI water to form a uniform solution. The pH value of solution was adjusted to six by NH_4_OH. Next, 0.4 g of TiO_2_ was added and sonicated for 1 h. The obtained suspension was transferred into a Teflon-lined autoclave and maintained at 160°C for 4 h. The precipitates were collected, followed by washing with 0.1 M of NaOH and DI water. The final products were dried in an oven at 60°C. Following the same process, photocatalysts prepared with different amount of CA (0.1, 0.15, 0.2, 0.25, and 0.3 M) were denoted as 0.1CD-TNs, 0.15CD-TNs, 0.2CD-TNs, 0.25CD-TNs, and 0.3CD-TNs, respectively.

### Characterization

X-ray diffraction (XRD) carves of samples were recorded using X-ray diffraction (XRD, Bruker AXS) with Cu Kα radiation. Fourier transform infrared spectrum (FTIR) results were characterized by a Thermo Nicolet IS10 spectrometer scanning from 4,000 to 500 cm^−1^. The microstructure of prepared samples was observed by transmission electron microscopy (TEM) and high-resolution transmission electron microscopy (HR-TEM) using a JEM-2010, JEOL instrument. UV-vis diffuse reflectance spectra (DRS) patterns were performed on a UV-3600 (Agilent, Cary300) instrument with BaSO_4_ as a reference. Photoluminescence (PL) spectra of liquid samples were performed by using an F-2500 spectrophotometer (Hitachi). X-ray photoelectron spectroscopy (XPS) data was achieved on a Thermo ESCALAB 250XI device. The degraded intermediates and final products of TC were detected *via* LC-MS (Waters, United States) using a BEH C18 column (1.7 um, 2.1*150 mm).

Electrochemical impedance spectroscopy (EIS) measurements were performed with a CHI-760 E electrochemical workstation (Shanghai Chenhua Instrument Co., Ltd, China), with a frequency range from 100 kHz to 0.1 Hz and an amplitude of the modulation signal was 5 mV. Photocurrent tests were measured using a conventional three-electrode system with 0.5 M of Na_2_SO_4_ as the electrolyte solution, Ag/AgCl as the reference electrode, and Pt wire as the counter electrode. The working electrode was prepared by spreading a slurry of the as-prepared photocatalyst onto the fluorine-doped tin oxide (FTO) glass.

For this study, congo red (CR), rhodamine B (RhB), and tetracycline (TC) were selected as water-soluble organic pollutants for photocatalytic degradation experiments. Visible light irradiation was provided by a 500 W (XO-CHX-ID) Xe lamp with a cutoff filter (λ≥ 420 nm) and the light intensity in the reactant center was around 25.6 mW/cm^2^. In a typical degradation procedure, 40 mg of catalyst was added into 40 ml of aqueous pollutants solution (CR, 120 mg L^−1^; RhB, 20 mg L^−1^; or TC, 80 mg L^−1^), and the solution was stirred for 30 min to acquire adsorption-desorption equilibrium. A small amount of the degraded solution was taken out at fixed intervals and centrifuged to remove the catalyst, then the UV-vis measurements were recorded for the supernatant.

## Results and Discussion

**FIGURE 1:**
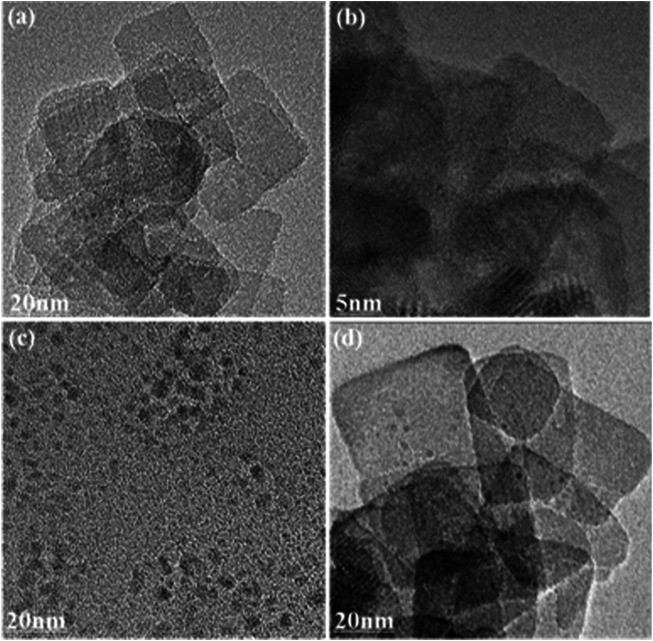
**(A)** TEM image of TNs; **(B)** enlarged TEM image of **(A)**; TEM images of CDs **(C)**; and 0.2CD-TNs **(D)**.

The typical TEM images of prepared TNs, CDs, and 0.2CD-TNs are shown in Figure 1. As shown in Figures 1A,B, TNs present a rectangular sheet-like structure and a lattice fringe of crystal, which can possess a large specific surface area. The TEM image of CDs (Figure 1C) displays a quasi-spherical morphology. Figure 1D shows that CDs disperse on the surface of TNs for 0.2CD-TNs, suggesting the coupling of CDs on TNs.

**FIGURE 2:**
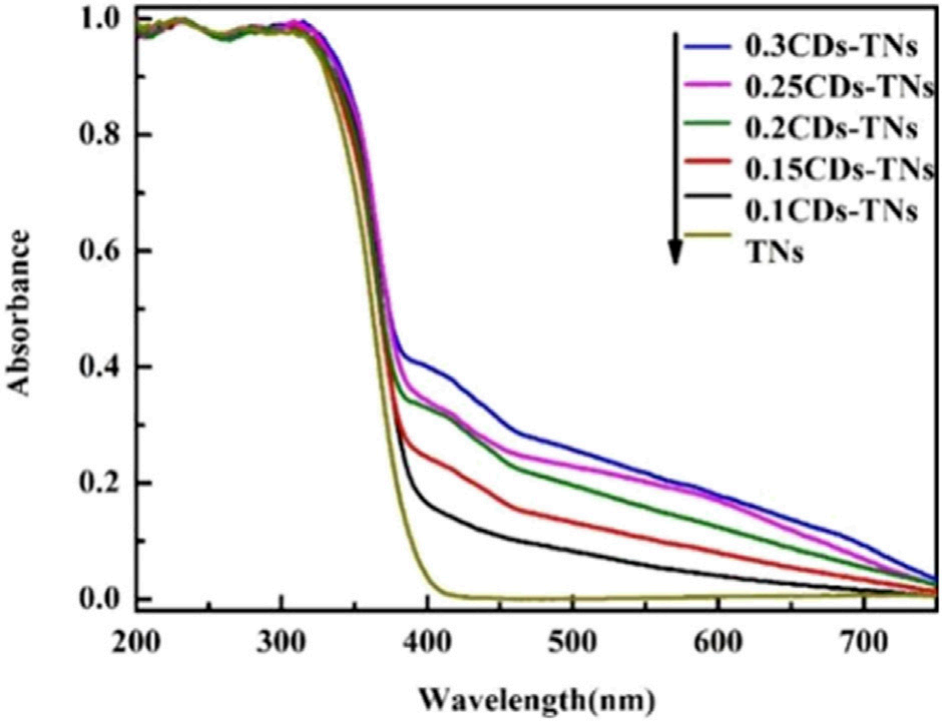
UV-vis DRS spectra of TNs and CD-TNs with different CDs content.

The light-harvesting capacity of photocatalyst is a vital factor for its photocatalytic performance (Shi et al., 2018; Valenti et al., 2016). To investigate the role of CD loading on the light absorption of TNs, the UV-vis diffuse reflectance spectra (DRS) of as-prepared samples are compared (Figure 2). For pure TNs, an absorption edge at about 400 nm is observed, exhibiting the bandgap of TNs is 3.2 eV, which is in accordance with previous reports (Li et al., 2018b). Compared to TNs, there is no obvious difference in the UV absorption after CD introduction. However, CD-TN photocatalysts reveal an enhanced visible light absorption with the increasing CD content, which directly confirms that the doping of CDs on TNs improves the visible light–harvesting capacity.

**FIGURE 3:**
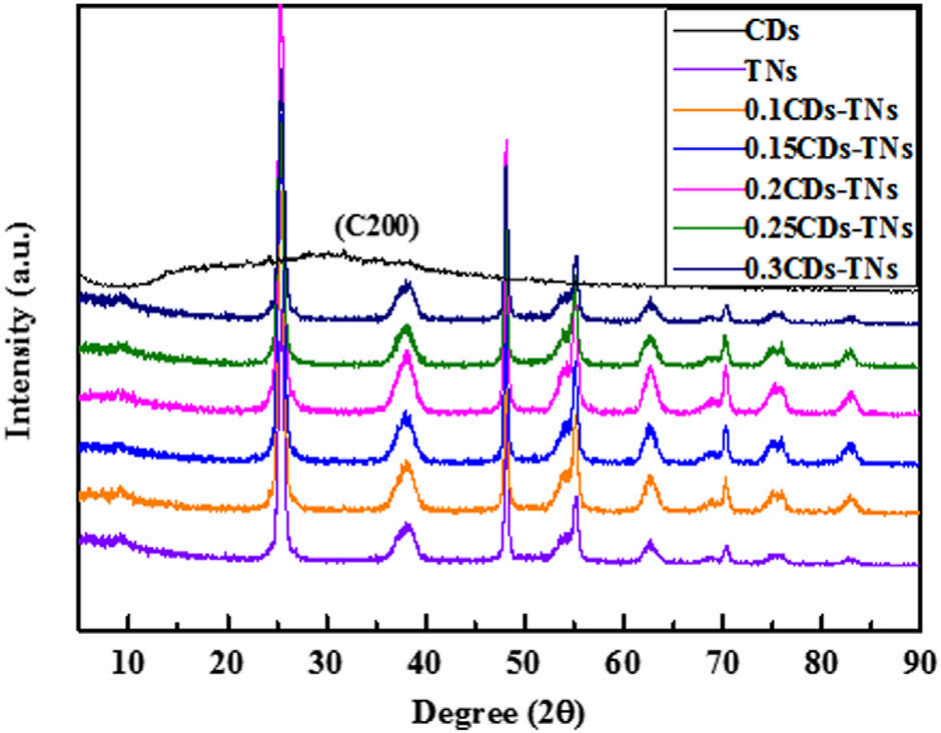
XRD patterns of CDs, TNs, and CD-TNs with different CDs content.

The crystal phase of synthesized samples was measured by X-ray diffraction (XRD) (Figure 3). The spectrum of CDs shows an obvious broad peak at around 29.7°, which is contributed to (200) planes of graphitic carbon (Qu et al., 2016). All the characteristic peaks in TNs and CD-TN photocatalysts are identical to the crystal planes of anatase TiO_2_ (PDF No.00–021-1272). Notably, no characteristic peak for CDs is detected in CD-TN photocatalysts with the amount of CDs from 0.1 to 0.3, which may ascribe to the relatively limited CD loading and low diffraction intensity in the composites (Shi et al., 2017). The peaks of CD-TNs with different CDs are consistent with TNs without any change, indicating that there is no obvious influence on the structure of TNs by loading CDs.

**FIGURE 4:**
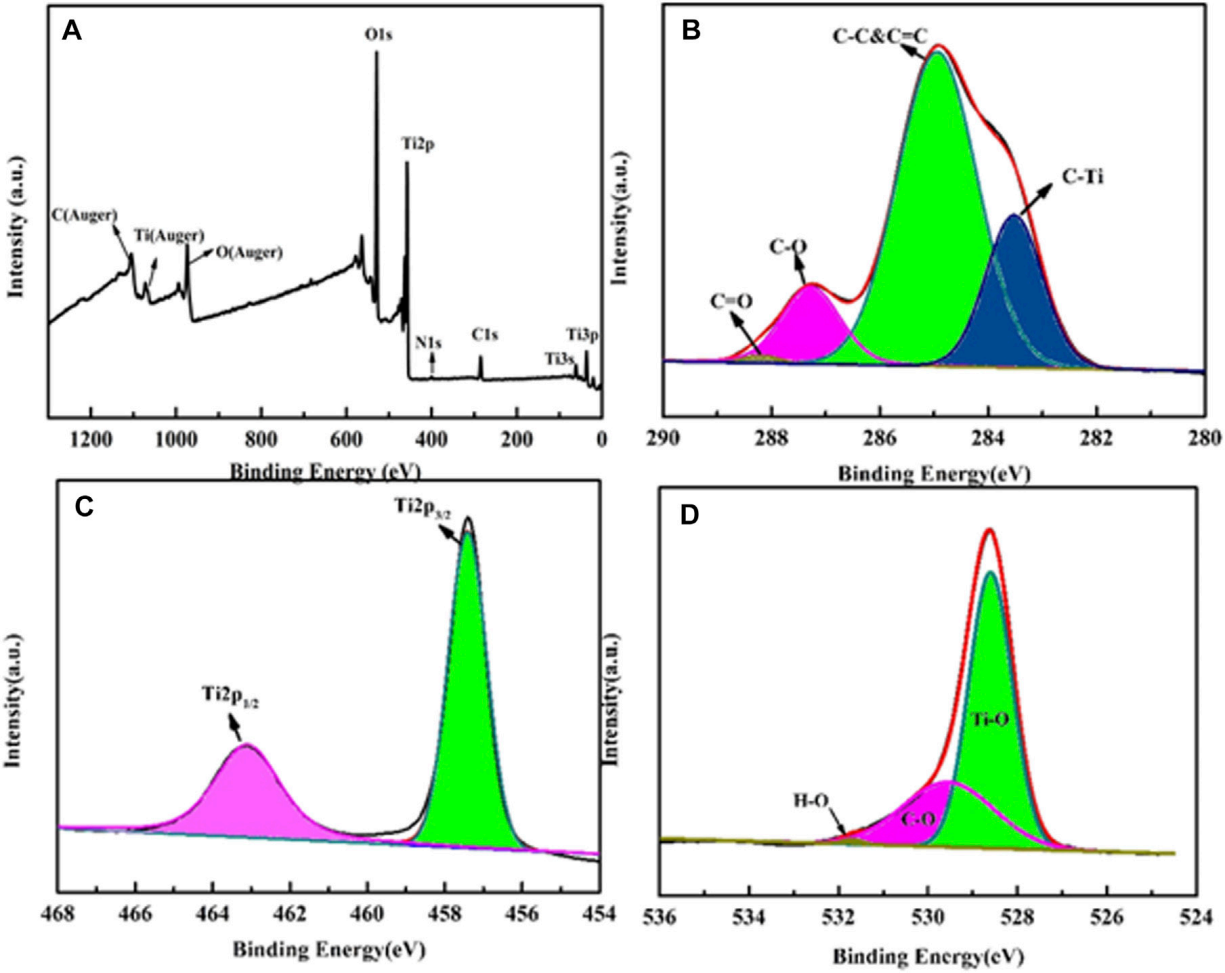
XPS spectra of 0.2CD-TNs: **(A)** survey XPS spectrum, **(B)** C1s, **(C)** Ti2p, and **(D)** O1s

The X-ray photoelectron spectroscopy (XPS) technique was conducted for CD-TNs to delve the valence band and the related elements, the results are illustrated in Figure 4. A survey scan (Figure 4A) depicts that the prepared CD-TN photocatalysts contain C, O, N, and Ti elements. Figure 4B shows the C1s spectrum, where the energies binding at 283.5, 284.9, 287.3, and 288.2 eV are attributed to C-Ti, C-C&C=C, C-O, and C=O, indicating the coexistence of CDs and TNs in the CD-TN catalysts. The Ti 2p spectrum shown in Figure 4C is deconvoluted into two signals: the bind energy observed at 457.4 eV is ascribed to Ti 2p_1/2_ and the peak at 463.1 eV comes from Ti 2p_3/2_. The O1s spectrum (Figure 4D) divided into three peaks at 528.6, 529.6, and 531.6 eV are corresponding to Ti-O, C-O, H-O, suggesting chemical interaction occurs between the two components.

**FIGURE 5:**
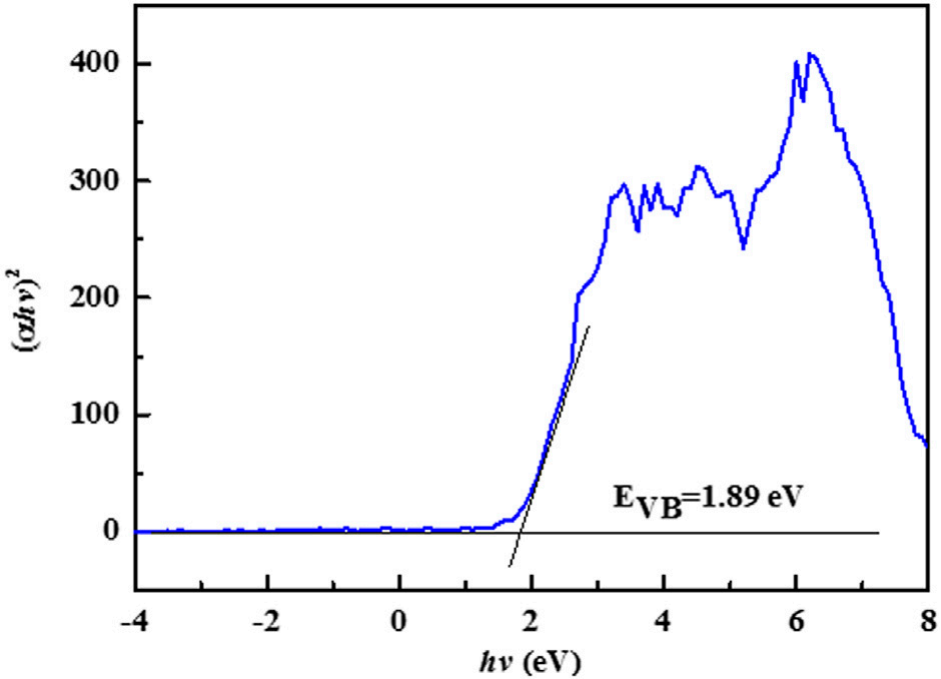
The bandgap energy of 0.2CD-TNs.

Meanwhile, based on the Tauc plot equation, the bandgap energy of the 0.2CD-TNs is estimated. As illustrated in Figure 5, the bandgap energy of 0.2CD-TNs is 1.89 eV, implying that prepared 0.2CD-TN photocatalysts can generate active species for degrading pollutants under visible light irradiation in theory.

**FIGURE 6:**
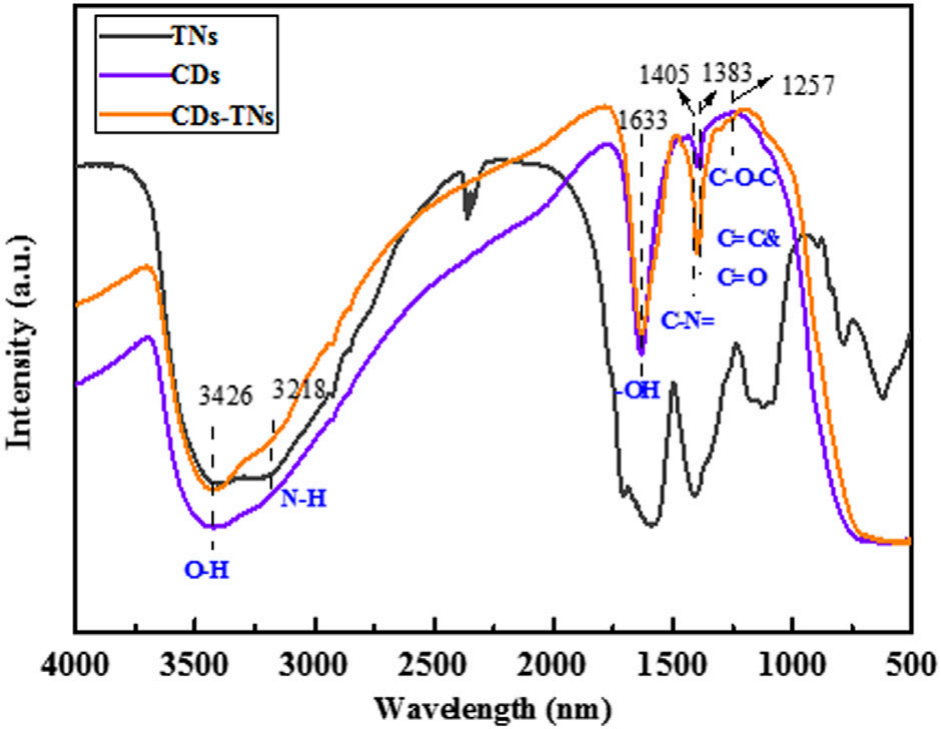
FTIR spectra of CDs, TNs, and 0.2CD-TNs.

The FTIR spectra of samples are shown in Figure 6. Three peaks emerged at 3,426, 1,633, and 1,383 cm^−1^ in all samples correspond to the stretching of O-H, C=C, and C=O, signifying the good hydrophilicity of CDs (Que et al., 2017). The peaks at 3,218 and 1,406 cm^−1^ in the FTIR spectrum of CDs are assigned to N-H and C-N = bonds, suggesting the presence of carboxylic and amide groups on the surface of CDs. In the FTIR spectrum of 0.2CD-TNs, a stronger peak centered at 1,383 cm^−1^ can be ascribed to the C=O group, and a new peak emerged at 1,257 cm^−1^ is assigned to the C-O-C bond. Furthermore, the characteristic peaks of CDs and TNs are detected, demonstrating that CDs are successfully introduced onto TNs.

**FIGURE 7:**
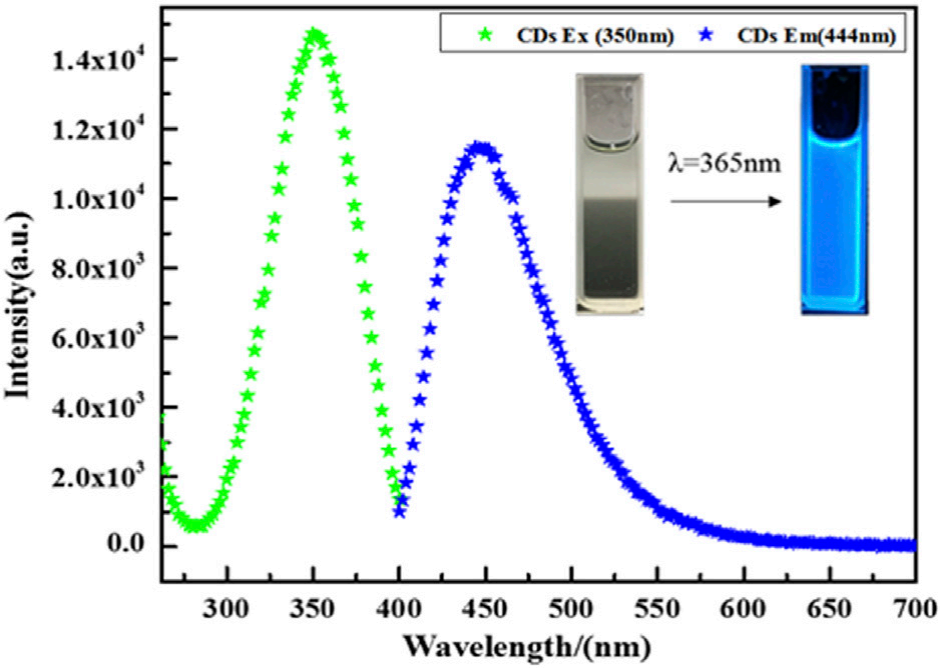
Upconverted PL spectra of CDs with excitation wavelength of 350 nm; inset: fluorescent picture of CDs under UV-light irradiation.

The photoresponse property of CDs is shown in Figure 7. When the excitation wavelength is 350 nm, the emission peak is located at about 440 nm. Significantly, CDs display bright fluorescence under UV-light irradiation, suggesting CDs possess the up-conversion property. CDs with the up-conversion property can absorb visible light and emit shorter wavelengths, which help CD-TNs utilize solar energy more efficiently.

**FIGURE 8:**
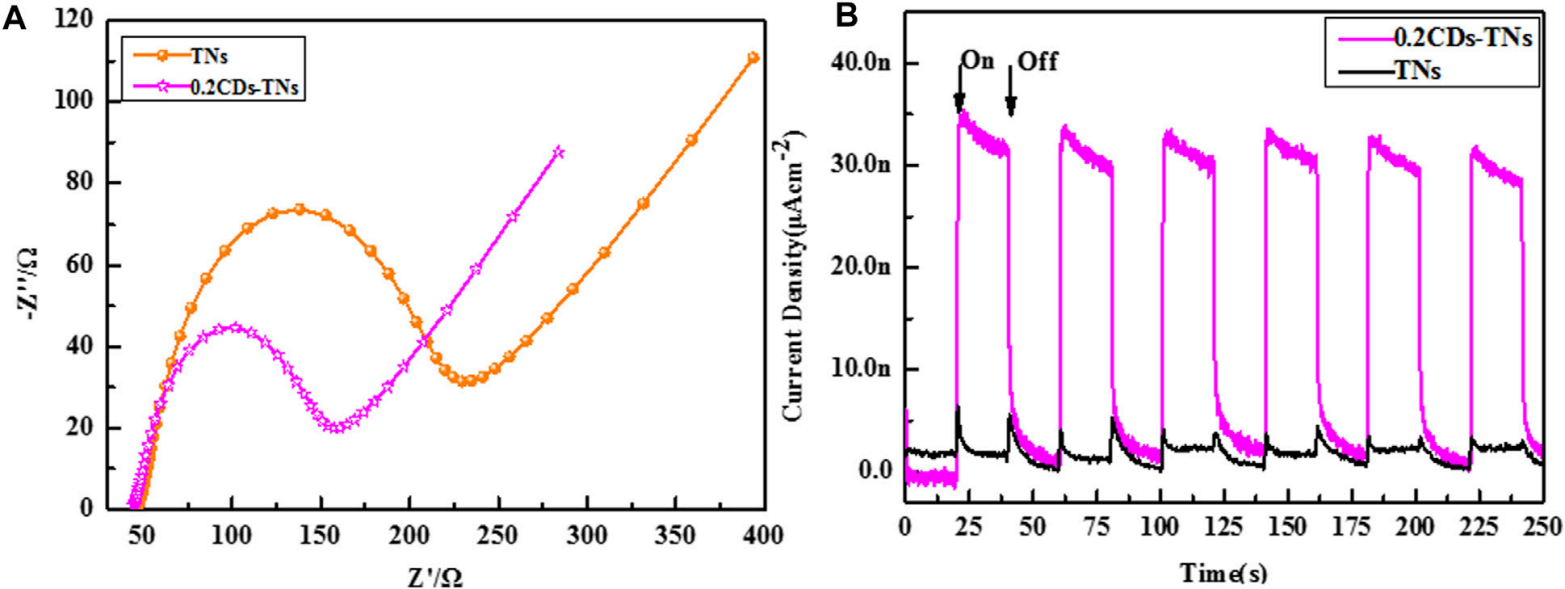
EIS curves **(A)** and transient photocurrent density **(B)** of TNs and 0.2CD-TNs under visible light irradiation.

For photocatalyst activity, the separation and transfer of photo-induced electron–hole in photocatalyst is considerably significant (Zhang et al., 2017c). Transient photocurrent and EIS are typical measurements used to delve the electron–hole excitation effect. As illustrated in Figure 8A, the arc radius of 0.2CD-TNs is narrower than that of TNs, indicating lower charge transfer resistance for 0.2CD-TN photocatalysts, which accelerates the charge transport at the interface of 0.2CD-TNs. Moreover, a uniform and increased photocurrent can be captured under light (Figure 8B), which verifies that CDs can effectively promote the charge transfer and migration, demonstrating a higher capacity in photocatalytic degradation of the CD-TN photocatalysts than that of TNs.

**FIGURE 9:**
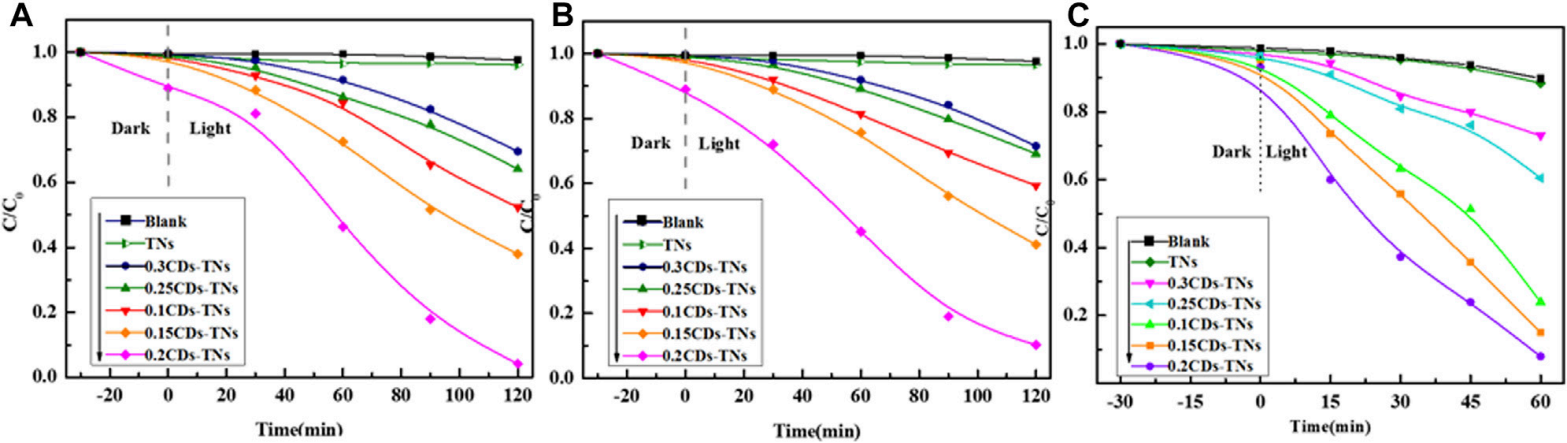
Photodegradation of **(A)** Congo red, 120 ppm, **(B)** RhB, 20 ppm, and **(C)** TC, 80 ppm by CD-TN composites with different contents of CDs under visible light.

The photocatalytic performance of prepared samples was tested *via* degradation of CR, RhB, and TC under simulated visible light irradiation (Figures 9A–C). It can be seen that in the presence of TNs, the concentration of CR, RhB, and TC changes negligibly under visible light. In contrast, CR, RhB, and TC are degraded remarkably in the same irradiation time in the presence of CD-TN photocatalysts. Additionally, the results reveal that the photocatalytic activity of CD-TNs greatly depends on the CD loading. The removal rate of CR is increased from 7.3 to 94.6% with the amount of CDs from 0 to 0.2 M. However, further increasing the amount of CDs from 0.2 to 0.3 M decreases the rate from 94.6 to 25.3%. Figures 9B,C depict the effect of CD-TNs on the removal of RhB and TC. The RhB degradation efficiency is measured to be around 97.2% under 150 min of irradiation in the presence of 0.2CD-TNs. Similarly, the TC degradation efficiency is achieved to be 96.1% for 0.2CD-TNs after 60 min of visible light irradiation. The experimental results also revealed that TC degrades fast because of the unstable fragments of TC (Supplementary Table S1, Supporting information). Among CD-TN catalysts, 0.2CD-TNs have the highest degradation efficiency, which probably contributes to that the high CD contents which will decrease catalytic active sites and enhance the charge carriers’ recombination rate (Giovannetti et al., 2015; Xie et al., 2018).

**FIGURE 10:**
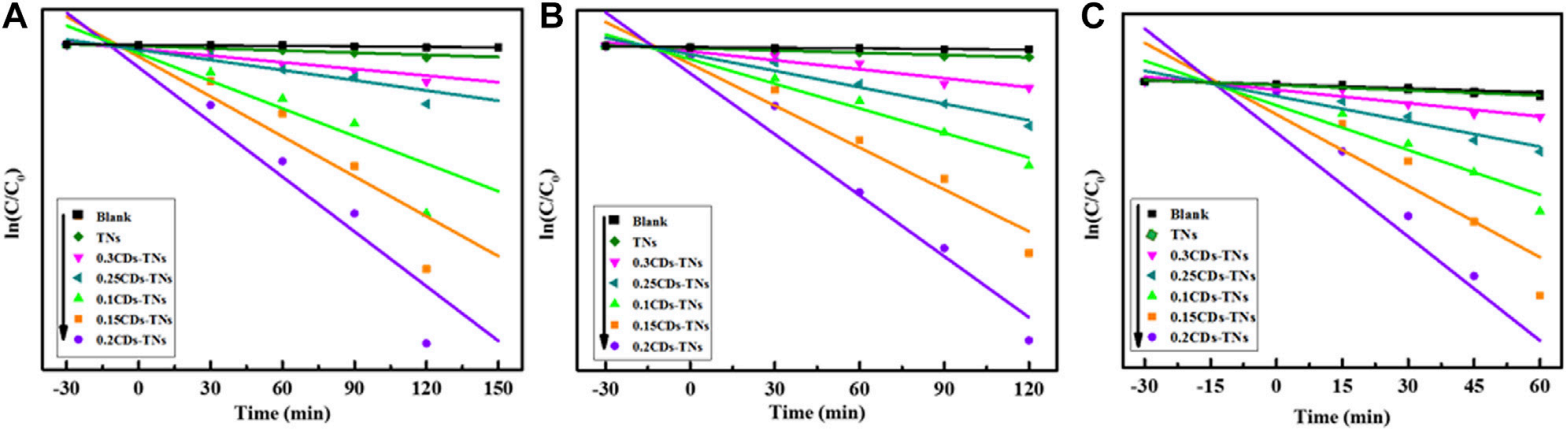
The degradation kinetics curves of **(A)** CR, **(B)** RhB, and **(C)** TC by CD-TN composites with different contents of CDs under visible light.

The kinetic fitting plots of ln (C/C_0_) against reaction time t for all photocatalysts are shown in Figures 10A–C. The results show that the degradation reactions of CR, RhB, and TC by CD-TN photocatalysts fit to pseudo first order kinetics. The formula can be expressed as follows:

$$\ln\left(C_{t}/C_{0}\right)=-kt,$$ (1)

where k is the kinetic rate constant, C_0_ is the initial concentration of pollutants, and C_t_ is the concentration of pollutants at different degradation time t. The parameters of photocatalytic degradation of CR, RhB, and TC by different photocatalysts are summarized in Table 1. For CR degradation, the degradation activity of 0.2CD-TN photocatalysts with k = 0.01409 min^−1^ displays 26.09 times higher than that of pure TNs. Furthermore, the k value of 0.2CD-TNs is much larger than other CD-TN photocatalysts under visible light, illustrating its outstanding capacity in pollutants removal. Moreover, similar trend can be observed for RhB and TC, and the degradation capacity for the three pollutant follows the sequence of 0.2CD-TNs >0.1 CD-TNs >0.15CD-TNs >0.25CD-TNs >0.3CD-TNs. It is apparent that 0.2CD-TNs show the highest reaction rate constants of 0.01542 and 0.02546 min^−1^ for RhB and TC, respectively.

**TABLE 1:** Degradation kinetic parameters of CR, RhB, and TC over TNs and CD-TN photocatalysts.

Pollutants	Samples	k (min^−1^)	*R* ^2^
CR	Blank	0.000153	0.89892
	TNs	0.00054	0.96714
	0.1 M CD-TNs	0.00568	0.9631
	0.15 M CD-TNs	0.00968	0.93802
	0.2 M CD-TNs	0.01409	0.94785
	0.25 M CD-TNs	0.00381	0.95356
	0.3 M CD-TNs	0.00208	0.91794
RhB	Blank	0.00014	0.82871
	TNs	0.000618	0.9828
	0.1 M CD-TNs	0.00778	0.93472
	0.15 M CD-TNs	0.01126	0.94741
	0.2 M CD-TNs	0.01542	0.95801
	0.25 M CD-TNs	0.00287	0.97289
	0.3 M CD-TNs	0.00187	0.96828
TC	Blank	0.00126	0.93376
	TNs	0.001	0.92232
	0.1 M CD-TNs	0.01092	0.87631
	0.15 M CD-TNs	0.01749	0.82157
	0.2 M CD-TNs	0.02546	0.84101
	0.25 M CD-TNs	0.00618	0.87702
	0.3 M CD-TNs	0.00326	0.89136

Besides, the BET surface area and total pore volume of 0.2CD-TNs are summarized. As shown in Table 2, the BET specific surface area and total pore volume of 0.2CD-TNs are 129.6286 m^2^/g and 0.244819 cm^3^/g, respectively, which can be attributed to the large surface area of TNs.

**TABLE 2:** Textural properties of 0.2CD-TNs.

Sample	BET surface area (m^2^/g)	Total pore volume (cm^3^/g)
0.2CD-TNs	129.6286	0.244819

**FIGURE 11:**
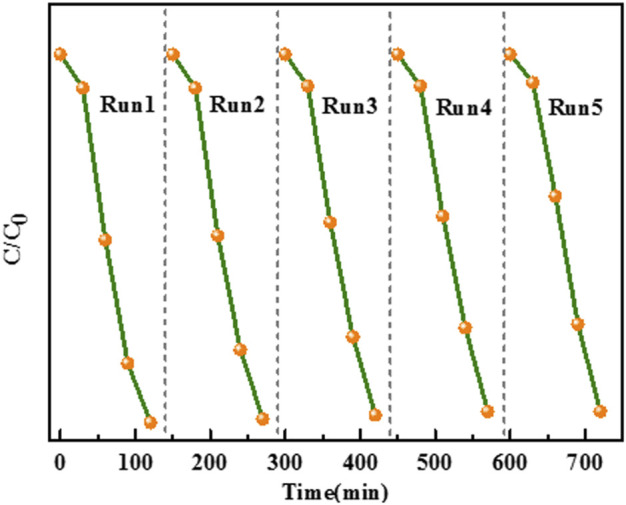
The stability of 0.2CD-TNs for Congo red removal.

The prospective applicability of CD-TNs for the practical stability was measured by degrading CR on the basis of the recycling experiments. As shown in Figure 11, after five recycling measurements, the CR degradation rate still remains at about 93.3%, and the photocatalytic activity of 0.2CD-TNs has no loss during the degradation process, indicating that the 0.2CD-TNs exhibit an excellent structural stability. Moreover, the XRD patterns of samples after reaction are shown in Supplementary Figure S1. It can be seen that there are no noticeable changes in the crystal structure for CD-TNs before and after degrading, which further verifies the robust stability of CD-TNs.

In Table 3, the photocatalytic performance of CD-TNs is compared with other catalysts reported previously. Obviously, taking into account the different experimental parameters, CD-TNs exhibit outstanding photodegradation activity among these catalysts.

**TABLE 3:** Comparison of CR, RhB, and TC degradation over different photocatalysts.

Pollutants	Photocatalyst	Light source	[Pollutants]	[Catalyst]	Removal (%)	Ref
CR	SCN/TiO_2_	Xe 300 W	50 mg/L	0.2 g/L	∼90 (1 h)	Zhang et al. (2017a)
CR	Co_3_O_4_/GO/TiO_2_	Xe 300 W	10 mg/L	0.25 g/L	∼87 (1.5 h)	[51]
CR	CD-TNs	Visible 300 W	120 mg/L	1 g/L	98.7 (2 h)	This study
RhB	GQDs/mpg-C_3_N_4_	Visible 300 W	10 mg/L	0.5 g/L	97 (2 h)	Li et al. (2018a)
RhB	g-C_3_N_4_/ZnTcPc/GQDs	solar light	20 μM	0.1 g/L	98.2 (1 h)	[47]
RhB	Bi_2_WO_6_	two EDMLs	10 mg/L	1 g/L	∼90 (1 h)	[55]
RhB	C-dots/g-C_3_N_4_	UV 3 W	10^−5^M	1 g/L	∼80	[56]
RhB	3DGA@CD-TNs	Visible 300 W	20 mg/L	1 g/L	97.6 (2 h)	This study (Li et al., 2018a)
TC	GQDs/mpg-C_3_N_4_	Visible 300 W	20 mg/L	1 g/L	65 (2 h)	[49]
TC	CDs-CoO	Visible 300 W	10 mg/L	0.5 g/L	87 (1 h)	[60]
TC	CeVO_4_/3DRGO/BiVO_4_	Xe 500 W	20 mg/L	0.5 g/L	∼90 (1 h)	Sordello et al. (2018)
TC	CDs/g-C_3_N_4_/MoO_3_	Visible 350 W	20 mg/L	0.6 g/L	88.4 (1.5 h)	[50]
TC	CQDs@In_2_S_3_/SWNTs	Xe 350 W	20 mg/L	0.8 g/L	∼90 (1 h)	
TC	3DGA@CD-TNs	Visible 300 W	100 mg/L	1 g/L	99.8 (40min)	This study

## Conclusion

Practical photocatalysts (CD-TNs) were synthesized *via* the hydrothermal method. The results show that all the CD-TN photocatalysts exhibit higher photocatalytic efficiency for pollutants degradation under visible light irradiation than that of TNs. For these samples, 0.2CD-TN photocatalysts present the highest reaction rate and more than 90% pollutants are removed after visible light irradiation. This excellent photocatalytic activity in CD-TN photocatalysts can be contributed to the merits of CDs that not only extend the light absorption but also impede the recombination and promote the charge transfer. Consequently, the results demonstrate that the CD-TN photocatalytic process is a promising technology for wastewater remediation and purification.

## Data Availability

The original contributions presented in the study are included in the article/Supplementary Material; further inquiries can be directed to the corresponding authors.
