# Supplementary material for: Carbon Dot–Doped Titanium Dioxide Sheets for the Efficient Photocatalytic Performance of Refractory Pollutants
Source: Front Chem. 2021 Sep 7;9:706343. doi: 10.3389/fchem.2021.706343 (PMC8453265; doi:10.3389/fchem.2021.706343)
Supplement: Supplementary file 1 [file DataSheet1.pdf]

Supplementary Materials

Carbon Dots doped Titanium Dioxide Sheets for the Efficient Photocatalytic Performance of Refractory Pollutants

Shen Shen [a,b], Rong Li[a,b], Hongbo Wang [a,b*], Jiajia Fu [a,b *]

[a] Jiangsu Engineering Technology Research Centre for Functional Textiles, Jiangnan University, No.1800 Lihu Avenue, Wuxi, P.R. China

[b] Key Laboratory of Eco-textiles, Ministry of Education, Jiangnan University, No.1800 Lihu Avenue, Wuxi, P.R. China

[*] Co-corresponding author; e-mail: wxwanghb@163.com
[*] Corresponding author; e-mail: kathyfjj@126.com

Table S1. Degradation intermediates of CR in the presence of 0.2CDs-TNs under solar light irradiation, detected by LC-MS in the positive ion mode.

Intermediate products	Retention time (min)	Theoretical mass (m/z)	Molecular Formula	Supposed structure
CR	4.13	696.68	$C_{32}H_{22}N_6Na_2O_6S_2$	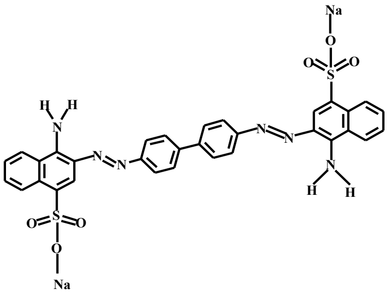
A	5.63 / 5.83	652.68	$C_{32}H_{22}N_6O_6S_2$	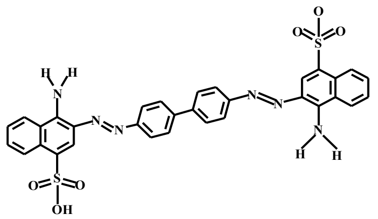
B	12.68	239.01	$C_{10}H_{10}N_2O_3S$	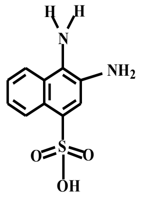
C	4.75	184.24	$C_{12}H_{12}N_2$	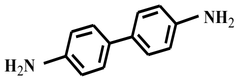
D	0.04 / 1.3	169.23	$C_{12}H_{11}N$	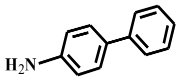
E	2.0 / 2.27	208.23	$C_{10}H_8O_3S$	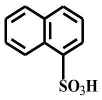
F	0.33	144.17	$C_{10}H_8O$	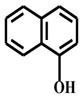

G	5.66	158.21	$C_{10}H_6O2$	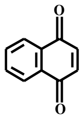 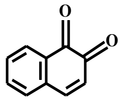
H	2.15 / 4.2	109.13	C_6H_7NO	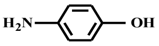
I	7.82	110.11	C6H6O2	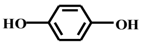 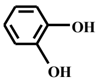 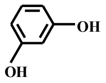
J	2.94	108.09	C6H4O2	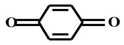
K	1.07	122.12	C7H6O2	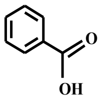
L	4.41	94.11	C6H6O	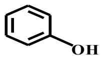
M	4.67	116.10	C6H6O3	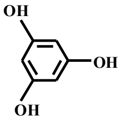

Table S2. Degradation intermediates of RhB in the presence of 0.2CDs-TNs under solar light irradiation, detected by LC-MS in the positive ion mode.

Intermediate products	Retention time (min)	Theoretical mass (m/z)	Molecular formula	Supposed structure
RhB	5.11	443.2335	$C_{28}H_{31}N_2O_3$	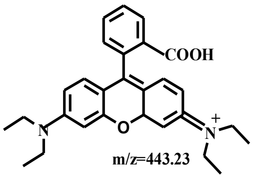
A	2.22	331.11	$C_{20}H_{15}N_2O_3$	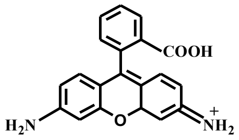

B	7.87 / 11.21	148.11	$C_8H_4O_3$	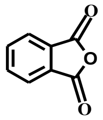
C	1.24 / 0.79	138.12	$C_7H_6O_3$	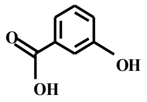
D	0.77	122.12	$C_7H_6O_2$	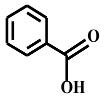
E	11.21	166.13	$C_8H_6O_4$	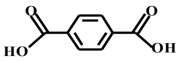 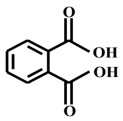
F	13.21	110.10	$C_6H_6O_2$	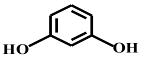 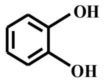 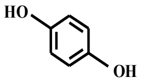
G	7.87	148.11	$C_5H_8O_5$	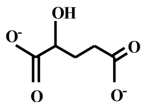
H	5.5 / 5.91	118.09	$C_4H_6O_4$	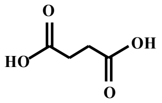
I	0.79	90.08	$C_3H_6O_3$	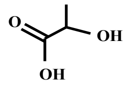
J	0.79	90.04	$C_2H_2O_4$	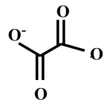

Table S3. Degradation intermediates of TC in the presence of 0.2CDs-TNs under solar light irradiation, detected by LC-MS in the positive ion mode.

Intermediate products	Retention time (min)	Theoretical mass (m/z)	Molecular formula	Supposed structure

TC	5.01	444.15	$C_{22}H_{24}N_2O_8$	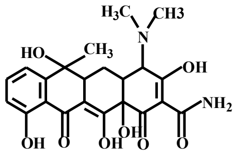 m/z=444.15
A	4.1 / 4.35 /4.5	443.15	$C_{22}H_{23}N_2O_8$	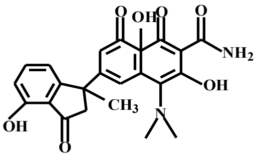
B	2.66	426.42	$C_{20}H_{22}N_2O_7$	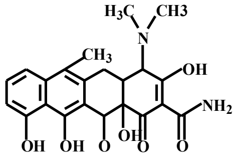
C	3.43	398.37	$C_{18}H_{18}N_2O_7$	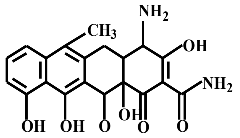
D	3.14 / 8.56	415.15	$C_{22}H_{20}N_2O_8$	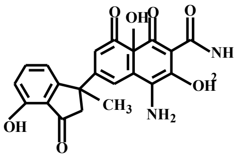
E	3.43	398.34	$C_{18}H_{16}NO_8$	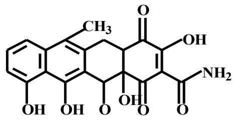
F	10.07	256.21	$C_{14}H_8O_5$	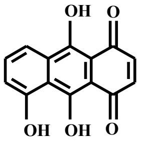
G	8.09 / 12.79	191.16	$C_{10}H_7O_4$	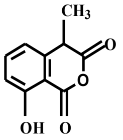
I	0.88	126.11	$C_6H_6O_3$	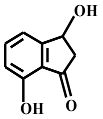
J	0.79	122.18	$C_7H_6O_2$	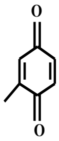
K	9.7	102.09	$C_3H_6N_2O_2$	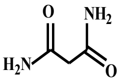

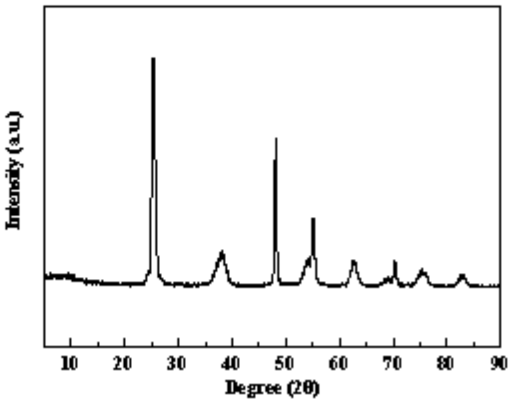

Figure S1 XRD carve of CDs-TNs after degrading experiments
